# Nicotinamide riboside or IL-17A signaling blockers to prevent liver disorders

**DOI:** 10.18632/oncoscience.338

**Published:** 2017-02-24

**Authors:** Ana Teijeiro, Nabil Djouder

**Affiliations:** Cancer Cell Biology Programme, Growth Factors, Nutrients and Cancer Group, Spanish National Cancer Research Centre, CNIO, Madrid, Spain

**Keywords:** NASH, HCC, NAD+, IL-17A, Nicotinamide riboside

Hepatocelular Carcinoma (HCC) is the fifth most common cancer and one of the most frequent aggressive liver neoplasms [1–3]. HCC has a poor prognosis and treatments are not very effective. Overall liver transplantation remains the best option for patients with HCC. Unfortunately, important limitations mainly due to scarcity of good-quality donor organs, high costs and immunosuppression problems restrict liver transplantation. Therefore, alternative treatments are considered to delay HCC recurrence, including resection, radiofrequency ablation and, chemotherapy with sorafenib, which can extend the patient lifespan of 2-3 months maximum. When recurrence occurs aggressive surgical treatment may have the best possible outcome [1].

The main risk factors for HCC enclose infection of hepatitis B and C viruses (HBV and HCV, respectively), toxin poisoning, chronic alcohol consumption and hypernutrition-associated obesity [3]. Nutrient overload leads to nonalcoholic fatty liver disease (NAFLD), characterized by fat accumulation in the liver or steatosis and considered as an important liver disorder arising in the obese population or in patients with type 2 diabetes (T2D) or hyperlipidemia [3]. Hepatic steatosis combined with low-grade inflammation and liver injury, triggers the development of nonalcoholic steatohepatitis (NASH) which can transit to HCC [3]. Thus, high body mass index increases HCC risks and overall cancer-related death [3]. Since obesity is reaching epidemic proportions, the absolute burden of NASH-related HCC is even higher than that of HCV/HBV-related HCC, therefore reaching a dramatic and alarming situation worldwide without any effective treatment.

NASH is often accompanied by several metabolic disorders including the metabolic syndrome comprising T2D [3]. Nutrient overload, through high insulin levels, deregulates hepatic functions, affecting the whole body metabolic balance leading to severe hepatic disorders. Nutrients can be inflammatory orchestrating NASH and HCC development [3]. We have recently demonstrated how nutrient excess causes immune responses and metabolic dysfunctions triggering hepatic fat accumulation, ultimately leading to NASH and HCC.

In this regard, we have generated a genetically engineered mouse model, hURI-tetOFF^hep^ mouse, in which, unconventional prefoldin RPB5 interactor (URI) is specifically expressed in murine hepatocytes inducing NASH and spontaneous HCC during a multistep process [2, 3]. Importantly, hepatic URI levels increase upon inflammatory cues, HBV infection or nutrient overload and thus, orchestrate NASH and NASH-induced HCC. HCC in hURI-tetOFF^hep^ mice covers HBV-associated human HCC signature [2, 3]. Unlike other models of NASH, such as methionine and choline deficient diet (MCD) or high fat diet (HFD) in which HCCs are not detected or, appearance is at very low incidence (about 4%), hURI-tetOFF^hep^ mouse recapitulates many features of human HCC and thus, represents an excellent model to study NASH and its progression to HCC. hURI-tetOFF^hep^ mouse also develops T2D-like phenotype, rendering this model very attractive to elucidate mechanisms of hepatic metabolic dysfunctions, NASH and HCC [3]. Recently, another mouse model recapitulating key features of human metabolic syndrome, NASH, and HCC was developed by long-term feeding of a choline-deficient HFD (CD-HFD) [4], representing an alternative elegant model of NASHinduced HCC.

hURI-tetOFF^hep^ mouse reveals histopathological features of human NASH including the presence of Mallory-Denk bodies, moderate steatosis, ballooned hepatocytes, immune cell infiltration, and liver injury [3]. Interestingly, hepatic URI expression is modulated by nutrient surpluses. Increased URI in hepatocytes inhibits aryl hydrocarbon (AhR) and estrogen receptor (ER). AhR and ER modulate L-tryptophan/kynurenine/ nicotinamide adenine dinucleotide (NAD^+^) metabolism and, their inhibition by URI decreases NAD^+^ levels in hepatocytes, thereby inducing DNA damage and initiating hepatic disorders. Thus, hURI-tetOFF^hep^ mouse mimics the nutrient overload model, both representing genotoxic stress models. Nicotinamide riboside (NR) a pyridinenucleoside form of vitamin B3 and precursor to NAD^+^ abolishes DNA damage in hURI-tetOFF^hep^ and in HFDfed mice [1–3]. Boosting NAD^+^ concentrations can also be therapeutic in certain metabolic disorders, such as T2D [5, 6] and fatty liver disease [7] and, potentially protects against obesity [8]. Importantly, boosting NAD^+^ by NR prevents HCC [1, 2]. Thus, NR may represent a preventive treatment for metabolic dysfunctions such as T2D, NASH and HCC.

DNA damage precedes hepatic inflammation and is essential to trigger T helper 17 (Th17) cells to the liver since NR treatment prevents T cell recruitment to livers of hURI-tetOFF^hep^ and HFD-treated mice [3]. Tryptophan is reported to promote Th17 differentiation in vitro. Hence, inhibition of kynurenine pathway possibly increases tryptophan concentrations supporting Th17 differentiation in the hURI-tetOFF^hep^ model. Secretion of IL-17A by Th17 cells generates systemic inflammation and promotes neutrophil recruitment to the white adipose tissue (WAT) thereby, leading to insulin resistance (IR) and lipolysis. Released free fatty acids are taken-up by the liver and stored as triglycerides in hepatocytes, leading to steatosis, liver injury and NASH. Inflammation precedes IR, steatosis and liver injury and, suggests that several hits are needed for NASH development, supporting the multi-hit hypothesis [3]. Several other studies suggest that inflammation is the driving force in NASH [3] and therefore, as demonstrated in the hURI-tetOFF^hep^ model, inflammation is determinant for the disease development. Steatosis per se may not account for the long-term prognosis of NASH.

Furthermore, IR seems to precede steatosis and NASH development suggesting that T2D is not a consequence of hepatic fat accumulation but T2D patients may progress to NASH and HCC. Abolishing genotoxicity with NR or blocking IL-17A signaling by anti-IL-17A antibodies or digoxin treatment (blocking Th17 cell differentiation) may provide an inexpensive, accessible and immediately translatable therapy for NASH and HCC, particularly in diabetic patients or individuals exposed at high risks.

In conclusion, our data identify mechanisms linking nutrient surpluses, inflammation, IR, NASH and HCC and, define genotoxic stress and IL-17A as the initiating and promoting players, respectively, in hepatic metabolic dysfunctions transiting to HCC [3]. These findings open doors to personalized medicine to prevent and cure NASH and NASH-induced HCC. We propose that patients at high risk of developing NASH (e.g. obese, T2D or HCVinfected patients) can be treated with NR, anti-IL-17A antibodies or digoxin preventing IR, steatosis, NASH and/ or HCC development (Figure).

**Figure 1:**
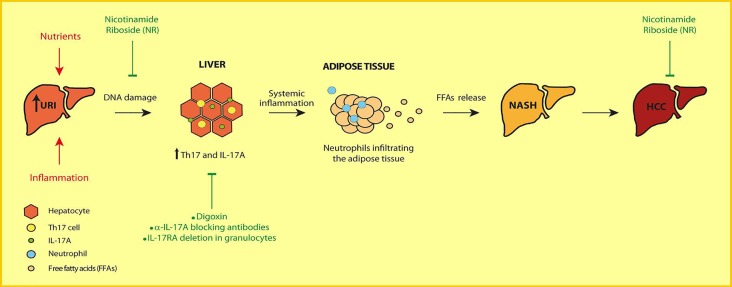
NR or IL-17A blockers against NASH-induced HCC development. Inflammatory cues or nutrient overload causes increased hepatic URI which, triggers DNA damage and orchestrates systemic inflammation-associated IL-17A ultimately inducing insulin resistance, hepatic lipid accumulation and NASH progressing to HCC.
